# Dissecting the molecular mechanisms of gene x environment interactions: implications for diagnosis and treatment of stress-related psychiatric disorders

**DOI:** 10.1080/20008198.2017.1412745

**Published:** 2018-01-17

**Authors:** Elisabeth B. Binder

**Affiliations:** ^a^ Department of Translational Research in PsychiatryMax-Planck Institute of Psychiatry, Munich, Germany; ^b^ Department of Psychiatry and Behavioral SciencesEmory University School of Medicine, Atlanta, GA, USA

**Keywords:** Gene x environment interaction, epigenetics, DNA methylation, early adversity, epigenética, metilación del ADN, estrés, trauma, interacciones gen x entorno, 表观遗传学, DNA 甲基化, 应激, 创伤, 基因 X环境交互

## Abstract

Epidemiological studies indicate a combined contribution of genetic and environmental factors, mainly exposure to adverse life events, in the risk for psychiatric disease. Understanding how adverse life events interact with genetic predisposition on the molecular level to shape risk and resilience to psychiatric disorders may yield important insight into disease mechanism. Using the example of the molecular mechanisms of interaction of functional genetic variants within the stress-regulating gene *FKBP5* and early adversity, it is delineated how this interaction could contribute to transdiagnostic disease risk via a combined genetic and epigenetic disinhibition of *FKBP5* transcription. This knowledge may now allow to develop biomarkers for a transdiagnostic subset of psychiatric patients and to personalize treatment.

Epidemiological studies indicate a combined contribution of genetic and environmental factors, mainly exposure to adverse life events, in the risk for psychiatric disease (Kendler, Davis, & Kessler, 1997; Kendler, Walters, & Kessler, 1997; Kessler, Davis, & Kendler, 1997). These two factors likely both converge to alter gene regulation and, in consequence, cell function. A central question that may help better understand risk and resilience to psychiatric disorder is how the environment (adverse life events) interacts with genetic predisposition on the molecular level to shape risk and resilience to psychiatric disorders. The identification of these mechanisms could then lead to biomarkers that would allow us to recognize individuals at high risk for early intervention strategies following exposure to adversity. It is clear that the complex interplay of multiple genes and diverse environmental factors likely leads to distinct underlying biological changes in patients with similar symptoms. Using this knowledge for an additional diagnostic sub-classification could support more personalized treatment strategies.

Exposures to adverse life events have been shown to acutely activate the stress hormone system and lasting effects of severe or chronic adversity on this system have been reported . The stress hormone system thus offers the possibility to study how environmental impact and genetic factors converge on the regulation of this system. When exposed to threat, this system is activated leading to the release of adrenocorticotropic hormone from the pituitary that then triggers glucocorticoid (GC) secretion from the adrenal gland (Heim, Newport, Mletzko, Miller, & Nemeroff, 2008). GCs are released in the systemic blood flow and reach every organ of the body, including the brain. They exert their effects through two types of receptors: the mineralocorticoid (MR) and the glucocorticoid (GR). Both are cytoplasmic receptors that translocate to the nucleus when activated. They serve as transcription factors and bind to specific DNA sequences, so called glucocorticoid responsive elements (GREs) that are enhancers or repressors of gene transcription. Via this mechanism, GCs can trigger a transcriptional response to stress that involves the up- or downregulation of a larger number of genes.

It is plausible that differences in the molecular response to GC activation are also associated with differences in the stress response not only on the cellular level, but also on the systemic and brain circuit level, with associated behavioural differences as well as differences in risk for stress- and trauma-associated psychiatric disorders. Indeed, common genetic variants alter the immediate transcriptional response to glucocorticoids (Arloth et al., 2015). These variants were mainly located in GREs, likely modifying the affinity of the DNA binding site to the receptor. These functional genetic variants were associated with risk for schizophrenia and depression when using data from large genetic meta-analyses from the Psychiatric Genomics Consortium. Exposure to adverse life events has been shown to increase risk for both disorders. Additionally, this set of stress-moderating genetic variants is associated with impaired learning of threat-related cues with inappropriately increased reactivity of the amygdala to neutral expressions in young adults. These findings suggest that genetic variants moderating the immediate cellular response to stress may also be associated with differences in stress-processing neural circuits and an increased risk for stress-related psychiatric disorders.

In addition to genetic differences, epigenetic variation can also influence the response to transcription factor. Epigenetic mechanisms modify the accessibility of the DNA to transcriptional regulators. They encompass post-translational modifications of histone proteins as well as chemical modifications of single nucleotides (most commonly in the form of methylation at cytosine guanine dinucleotides [CpGs] = DNA methylation). More condensed chromatin would thus be more prohibitive for binding of GRs and transcriptional regulation of target genes. A series of studies have shown that environmental exposure, especially adversity early in life, can lead to lasting changes in gene function by inducing such epigenetic changes (Fiori & Turecki, 2016; Klengel & Binder, 2015). Exposure to adversity was shown to lead to changes in DNA methylation in both peripheral tissues and post-mortem brains, with complex patterns of increased and decreased DNA methylation emerging with exposure, depending on the region in the genome. Some of these environmentally induced changes may be reversible, although more research is needed. It is likely that a series of different mechanisms will drive these changes, some very specific for certain cell types and brain circuits and likely linked to specific experiences and adaptive coping mechanisms, others more global and possibly linked to system wide effects of the stress response (hormones, catecholamines, immune system). Congruent with the latter type of mechanisms, direct effects of the stress hormone glucocorticoids on the epigenome have been described. GR activation has been shown to induce lasting epigenetic changes at GRE by locally decreasing the level of DNA methylation. This DNA demethylation of GREs subsequently facilitates the transcriptional effects of the GR on the target genes (Kress, Thomassin, & Grange, 2001, 2006). Excessive glucocorticoid release after stress exposure may thus induce long-lasting epigenetic changes and, by this, contribute to the biological embedding of risk trajectories and could sensitize an individual’s response to subsequent stress exposure.

It is also likely that these epigenetic responses to GC are in turn moderated by genetic variants, either by changing the activity or the binding of the GR itself, or the accessibility to molecules that would promote and stabilize such epigenetic changes. The following example will illustrate how genetic and epigenetic changes can moderate and possibly mediate adversity-associated risk for psychiatric disorders.

FKBP5 as an example for mechanisms of gene x environment interactions in psychiatry:

FK506 Binding Protein-5 is encoded by the gene *FKBP5*. Within the cell, *FKBP5* plays a central role in the stress response. It is part of the GR complex (Grad & Picard, 2007). When FKBP5 is bound to the complex, the GR has low affinity to cortisol and does not translocate readily to the nucleus (Davies, Ning, & Sanchez, 2002; Wochnik et al., 2005). *FKBP5* is also a target of GR activation, and its mRNA and protein are induced by cortisol. Within a few hours, *FKBP5* is strongly upregulated (5–10 fold) by stress or glucocorticoids in a number of tissues, including the brain (Menke et al., 2012; Scharf, Liebl, Binder, Schmidt, & Muller, 2011). This creates an ultra-short negative feedback loop, whereby GR activation induces *FKBP5*, which then limits GR activity by binding to the GR-complex (Vermeer, Hendriks-Stegeman, van der Burg, van Buul-Offers, & Jansen, 2003). However, FKBP5 not only regulates the function of the GR, but also many other proteins and pathways that are implicated in neuronal function and synaptic plasticity, such as the mTOR and calcineurin pathways but also autophagy and DNA methyl-transferases (Rein, 2016). *FKBP5* would thus serve as a molecular amplifier of the stress response, able to influence a number of downstream pathways, likely in a cell-type specific manner.

Any changes in this upregulation, be it via genetic or epigenetic factors, could thus have important influences on stress-related behaviour. Animal studies show that increased expression of *FKBP5* (especially in the amygdala and the hippocampus) is associated with decreased stress coping behaviour, increased anxiety and impaired extinction learning (see Zannas, Wiechmann, Gassen, & Binder, 2016, for review).

On the genetic level, this stress-related induction of *FKBP5* mRNA is moderated by common genetic variants (tagged by the functional single nucleotide polymorphism rs1360780) in the FKBP5 locus (Binder et al., 2004; Klengel et al., 2013). Most humans (about 65% as seen in data from the 1000 genomes project) carry the allele that is associated with a moderate increase in *FKBP5* following GR activation; 35% carry the allele that leads to a more exaggerated mRNA response. These genetic effects on the molecular stress regulation are reflected on the endocrine, behavioural and imaging level. First, the changes in GR sensitivity during the feedback regulation of the HPA axis lead to prolonged stress-related cortisol release in individuals carrying the variant that is associated with higher *FKBP5* mRNA induction (Buchmann et al., 2014; Ising et al., 2008; Luijk et al., 2010). Second, the high induction allele is associated with different behavioural responses to threat and trauma such as increased dissociation following trauma, increased bias towards threat and increased intrusions (Cheung & Bryant, 2015; Fani et al., 2013; Koenen et al., 2005). Third, this variant is also associated with both structural as well as functional changes in brain imaging with increased hippocampal and amygdala activity to threat and white matter abnormalities in the posterior cingulum in high expression allele carriers (Fani et al., 2013, 2014, 2016; Hirakawa et al., 2016; Holz et al., 2015). Overall, these human genetic studies thus agree that high FKBP5 expression is associated with a similar phenotype profile as described in animal studies, including an increased bias towards threat, increased intrusions and delayed normalization of the stress response following psychosocial stressors.

This genetic change in the endocrine, circuit level and behavioural stress response is associated with an altered risk for stress-related psychiatric disorders. Interactions between FKBP5 and stressful life events, especially those occurring in childhood, were found to be associated with a variety of psychiatric disorders, including PTSD, depression, aggression, suicidality and psychosis in a large number of studies including well over 18,000 individuals (for reviews, see Halldorsdottir & Binder, 2017; Zannas et al., 2016). Genome-wide association studies and candidate gene case-control association studies have not found a main effect for this gene in predicting psychopathology, indicating that the effect is dependent on environmental influences. This suggests that additional mechanisms other than the genetic regulation of *FKBP5* are necessary to unveil the stress interaction effect.

In the case of *FKBP5*, genetic and epigenetic changes need to come together. Specifically, changes in DNA methylation of *FKBP5* locus glucocorticoid response elements have been implicated that lead to an enhancement of the transcription response of *FKBP5* to GC (Klengel & Binder, 2013; Klengel et al., 2013). Only the combination of risk allele carrier status and exposure to child abuse is associated with a reduction in DNA methylation at these response elements. These effects have been observed in DNA from peripheral blood cells and saliva in both adults exposed to early trauma as well as children (Klengel et al., 2013; Non et al., 2016; Tyrka et al., 2015; Yehuda et al., 2016). One possible mechanism here could be that the genetic variants lead to increased *FKBP5* expression following GR activation, delaying the negative feedback phase and resulting in prolonged cortisol response to stress and trauma. This in turn would then precipitate GR-induced DNA demethylation at GREs in *FKBP5* and a transcriptional disinhibition (Klengel & Binder, 2013). Direct exposure of a hippocampal neuronal progenitor cell line to GC reveals decreased DNA methylation at the same sites that show reduced methylation in peripheral blood in individuals exposed to early trauma and carrying the high-induction allele of *FKBP5* (Klengel et al., 2013).

This disruption in regulatory homeostasis of *FKBP5* following stress, caused by both a genetic and an epigenetic disinhibition, could result in long-lasting changes in the neural circuits involved in stress and anxiety regulation. This could be driven by changes in GR tone but also by downstream effects of *FKBP5* on additional pathways that are relevant for neuronal function and synaptic plasticity. As mentioned above, early trauma x FKBP5 genotype interactions predict risk for a range of psychiatric disorders. It could be possible that patients suffering from such an *FKBP5* disinhibition, but presenting with distinct psychiatric symptoms, could benefit from blocking FKBP5 activity. A selective high-affinity antagonist for *FKBP5* has been developed, and first experiments in experimental animals show that is has anxiolytic effects and increases stress coping (Gaali et al., 2015; Hartmann et al., 2015).

Dissecting the molecular mechanisms underlying the statistical gene x early trauma association of *FKBP5* has identified a combined genetic and epigenetic disinhibition of stress-induced *FKBP5* transcription as a possible underlying cause of disease risk. This knowledge may now allow the development of biomarkers for this transdiagnostic subset of psychiatric patients and to personalize treatment. This could include selective inhibition of *FKBP5* but also targeted psychotherapy approaches for the underlying risk behaviours such as an increased bias towards threat or increased intrusions observed with increased *FKBP5* function (see Figure 1). A study by Wilker et al. shows that *FKBP5* genotype moderates long-term effectiveness of exposure-based psychotherapy for posttraumatic stress disorder, with patients carrying the genotype associated with higher *FKBP5* function showing increase relapse rates at 10 months (Wilker et al., 2014). Knowledge about a patients *FKBP5* status could thus help guide the type of treatment as well as the frequencies of follow-up visits. The next step will be to expand this set of biomarkers and mechanistic insights to genome-wide approaches.

**Figure 1.**
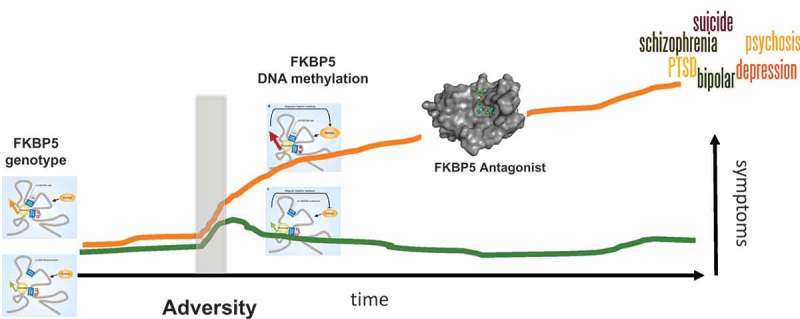
How *FKBP5* genotype may shape risk vs. resilience to exposure to adversity during childhood. In the light of adversity, the genotype effect is unmasked and needs additional epigenetic changes at the FKBP5 locus to occur. This leads to a transcriptional disinhibition of *FKBP5* and high FKBP5 levels, associated with increased anxiety and decreased stress coping. A combined *FKBP5* risk genotype and exposure to childhood trauma has been associated with risk for a number of psychiatric disorders, suggesting that patients with high *FKBP5* could represent a transdiagnostic subtype of patients who could benefit from common treatment strategies, such as *FKBP5* antagonists.
